# Case Report: TAFRO syndrome misdiagnosed as liver cirrhosis due to persistent abdominal distension

**DOI:** 10.3389/fimmu.2025.1554198

**Published:** 2025-04-03

**Authors:** Luyuan Ma, Ruolan Gu, Luzhuo Ma, Xiaoting Li, Lan Yang, Peng Xie, Xuelan Xiao, Feng Gao, Fang Liu, Chuan Shen, Caiyan Zhao

**Affiliations:** ^1^ Department of Infectious Diseases, Hebei Medical University Third Hospital, Shijiazhuang, China; ^2^ College of Basic Medicine, Chengde Medical University, Chengde, China; ^3^ Department of Nuclear Medicine, Hebei Medical University Third Hospital, Shijiazhuang, China; ^4^ Department of Pathology, Hebei Medical University Third Hospital, Shijiazhuang, China

**Keywords:** TAFRO syndrome, liver cirrhosis, abdominal distension, misdiagnosis, ^18^F-FDG PET/CT

## Abstract

This report described a male patient presenting with recurrent fever, fatigue, persistent abdominal distension, and diarrhea, who was repeatedly misdiagnosed with decompensated cirrhosis at multiple hospitals. The diagnostic process was complex, ultimately leading to the diagnosis of TAFRO syndrome through lymph node and bone marrow biopsies, following ^18^F-FDG PET/CT imaging. The patient initially responded to treatment, while later succumbed to severe intra-abdominal infection and multiple organ failure. TAFRO syndrome, a rare subtype of Castleman disease, is frequently misdiagnosed due to limited clinical awareness, delaying appropriate treatment. This case highlighted the diagnostic challenges and therapeutic considerations associated with TAFRO syndrome, providing insights for clinical practice based on the existing research.

## Introduction

TAFRO syndrome is a rare systemic inflammatory disease characterized by fever, anasarca, thrombocytopenia, reticular fibrosis (or renal insufficiency), and organ enlargement with nonspecific clinical manifestations, which was initially proposed in Japan in 2010 (1). TAFRO syndrome can involve multiple organs, including the liver, kidneys, intestines, and lymph nodes, and is highly susceptible to misdiagnosis. Early diagnosis and prompt treatment are crucial for reducing its mortality rate. Physicians specializing in hematology, gastroenterology, and nephrology should develop a deep understanding of its clinical manifestations and diagnostic criteria. TAFRO syndrome is classified as a subtype of idiopathic multicentric Castleman disease (iMCD), significantly varying from the classic form. The majority of patients present with a subacute onset and a progressively worsening clinical course. Insufficient immunosuppression can lead to disease progression, mainly resulting in bleeding and multi-organ failure, whereas excessive immunosuppression increases the risk of severe infections and drug-related adverse events. In this study, a patient severe TAFRO syndrome, who was initially misdiagnosed as cirrhosis accompanied by multiple abdominal complications, was described. Despite treatment efforts, the patient exhibited minimal improvement, and the final diagnosis of severe iMCD-TAFRO was confirmed through a multidisciplinary approach. Although individualized chemotherapy was initiated, the disease progressed, and adverse drug reactions ultimately resulted in multi-organ failure. A deeper understanding of the clinical characteristics and pathological changes of iMCD-TAFRO can enhance the ability of non-hematologists to diagnose the disease, facilitate timely diagnosis and treatment, and ultimately reduce mortality.

## Case presentation

A 58-year-old man presented to the hospital with a six-month history of recurrent fever, accompanied by persistent abdominal distension and fatigue for one year. The fatigue and abdominal distension had persisted over the past six months, and body temperature normalized spontaneously. Initially, the patient experienced mild fatigue and persistent abdominal distension, along with unexplained fever (Tmax 39.0°C), predominantly in the afternoon. However, there were no accompanying symptoms, such as cough, nausea, vomiting, or abnormalities in urine and stool. Despite seeking medical attention at two hospitals, no definitive diagnosis was established. Six months later, laboratory tests revealed thrombocytopenia (PLT 72.00×10^9^/L), renal insufficiency (CREA 1.44mg/dL), and elevated inflammatory markers C-reactive protein (CRP) 25.28 mg/L, erythrocyte sedimentation rate (ESR) 85 mm/h. Abdominal imaging demonstrated significant ascitic fluid accumulation, and biochemical and routine analysis of the ascites revealed an abdominal infection. However, no specific pathogen was identified. The patient’s abdominal symptoms recurred frequently, while exhibited temporary relief with anti-inflammatory therapy, repairs the liver cells, diuretics, ascites drainage, and albumin supplementation. During multiple screenings of immune-related indicators, serum protein electrophoresis revealed an elevated γ-globulin fraction (41.6%). Immunoglobulin levels were as follows: IgG 14.25 g/L (1-4.2g/L), IgA 3.82 g/L (8.6-17.4g/L), IgM 0.65 g/L (0.3-2.2g/L), and IgG1 11.6 g/L (4.05-10.11g/L), and no other significant abnormalities were identified. The patient was subsequently admitted to the Department of Infectious Diseases for further evaluation and treatment. Upon assessment, a weight loss of 20 kg was noted. The primary clinical manifestations included frequent episodes of yellow, loose stools, occurring approximately six times per day.

### Physical examination

The patient appeared chronically ill and thin, accompanying with visible liver palms and spider angiomata on the face. Lymphadenopathy was detected in the neck, axillae, and groin. Abdominal distension was evident, along with prominent abdominal wall varicosities. The liver was palpable 4 cm below the costal margin, and the spleen extended 10 cm below. Shifting dullness was positive, and mild pitting edema was found in both lower extremities.

### Diagnosis and treatment process

**Figure 1:**
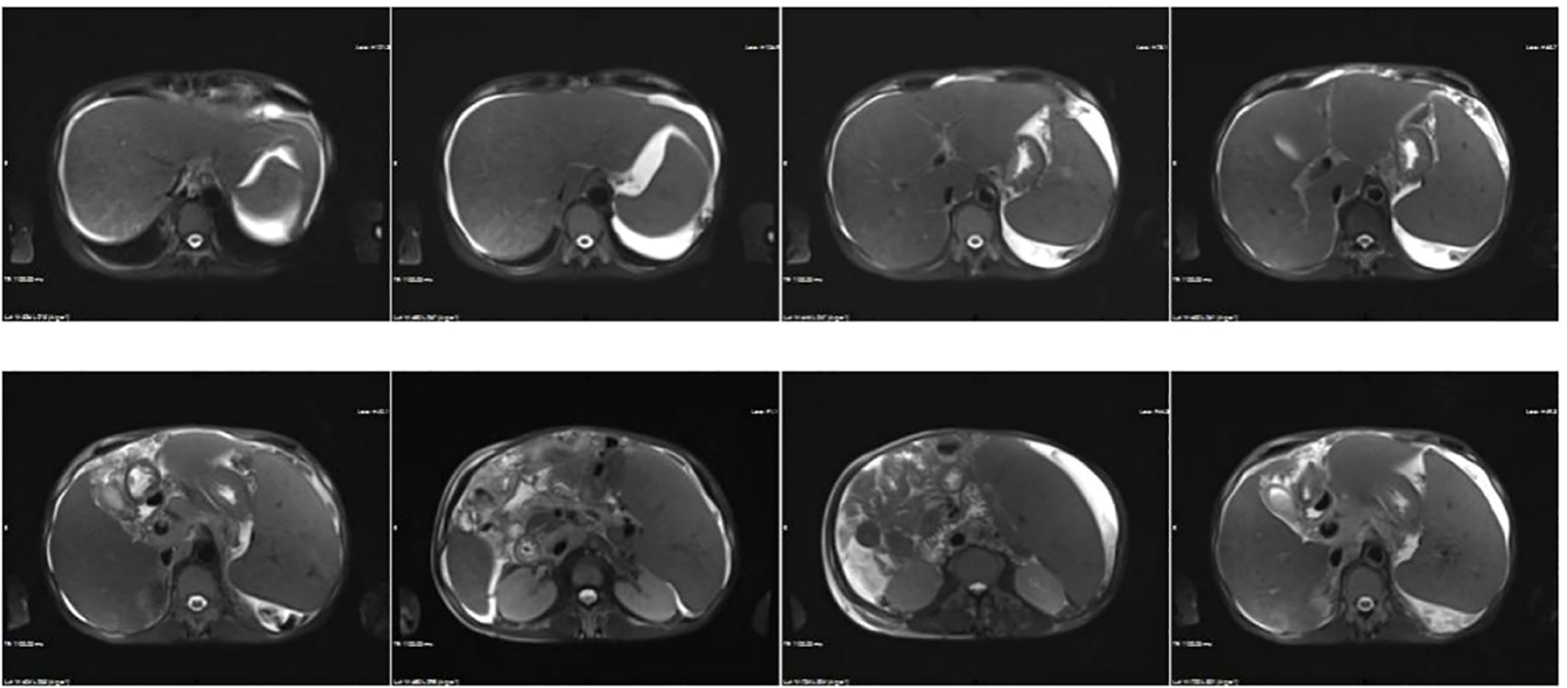
Abdominal MRI displaying heterogeneous liver signals, suggestive of possible cirrhosis and portal hypertension. Additional findings include cholecystitis with cholestasis, splenomegaly, ascites, and intestinal tube abnormalities.

Blood tests indicated a reduction in hemoglobin and platelet levels compared with pre-admission values (HGB 90 g/L, PLT 56 × 10^9^/L). Results of liver disease-related laboratory tests were unremarkable including autoantibodies and hepatotropic virus, (**Supplementary Table**). Renal function remained stable (CREA 1.48mg/dL, UA 746 μmol/L). Routine urine tests indicated occult blood 3+ and protein +. Kidney disease indicators included urine IgG (33.4 µg/ml), microalbumin (46.7 µg/ml), blood β2-microglobulin (6.03 µg/ml), urine β2-microglobulin >10 µg/ml, 24-hour urine protein (1.2 g/L), and glycosylated hemoglobin (6.5%). Coagulation function were abnormal, and prothrombin time (PT) 19.9s, prothrombin activity (PTA) 44%, international normalized ratio (INR) 1.78, activated partial thromboplastin tim (APTT) 48.7s, D-Dimer 6.11ug/ml, Fibrinogen degradation products (FDP) 30.29ug/ml. Inflammatory markers’ levels were elevated, and procalcitonin (PCT) 2.12 ng/mL, CRP 9.50 mg/L, IL-6 94.52 pg/mL, and ESR 51 mm/h exhibited the increased levels. Stool analysis revealed unformed loose stools without other abnormalities; a stool smear indicated 70% cocci, while no specific pathogen was detected. Abdominal imaging revealed ascites, portal hypertension, and pericardial effusion. The above results indicated the possibility of abdominal disease, and then the MRI and MRCP of the abdomen (**Figure 1**) were conducted, which indicated liver cirrhosis, portal hypertension, cholecystitis with cholestasis, ascites, and splenomegaly. Ascites white blood cell (WBC) at 1330×10^6^/L, 91% monocytes, and 9% multinucleated cells, but the culture was negative. The diagnosis included ascites, hypoproteinemia, intestinal dysbiosis, renal insufficiency. Treatment involved albumin, terlipressin, furosemide, spironolactone, tolvaptan, rifaximin, probiotics. Despite comprehensive treatment, the patient’s symptoms exhibited minimal improvement.

**Figure 2:**
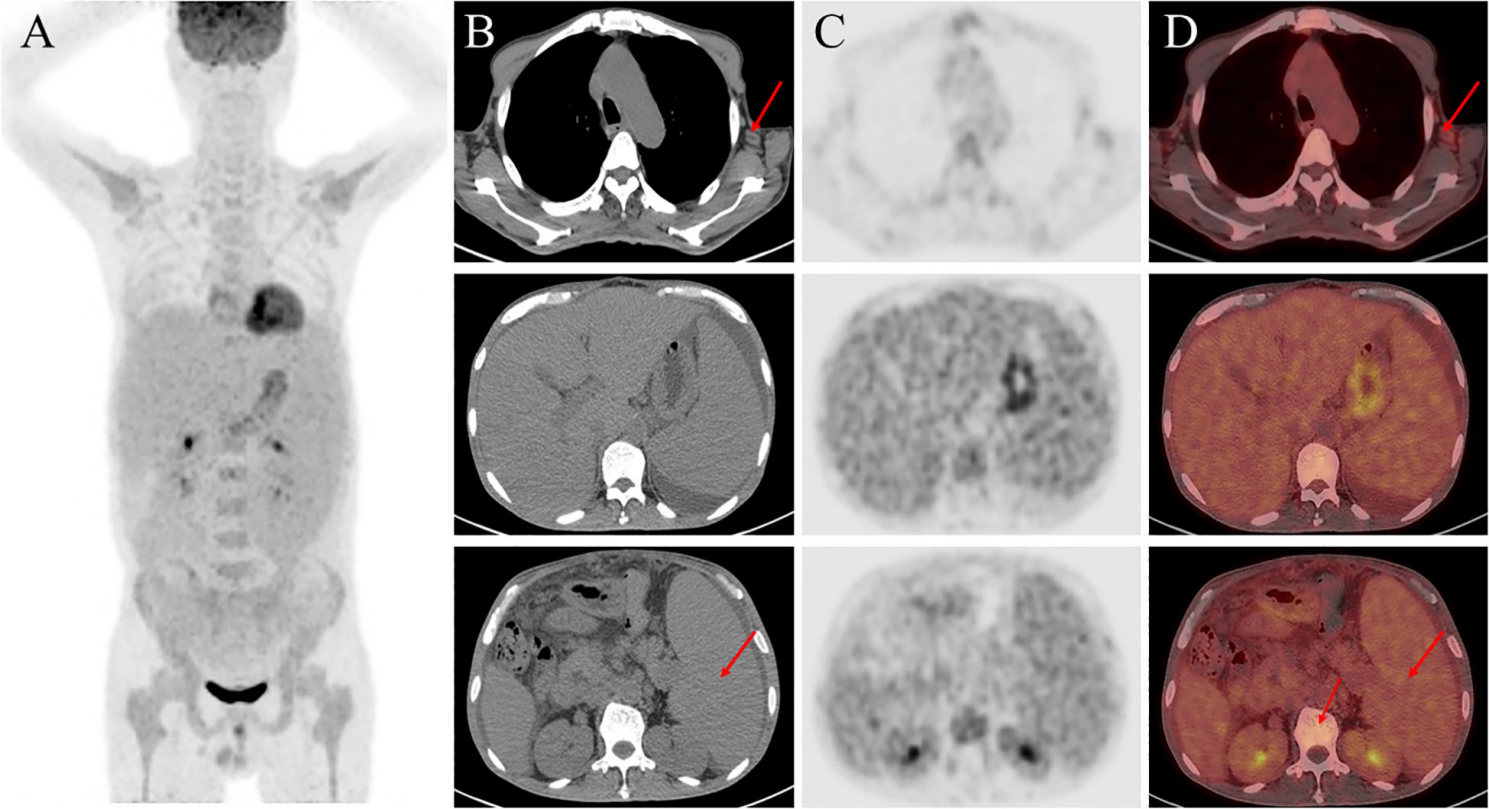
^18^F-FDG-PET/CT imaging. **(A)** Maximum intensity projection (MIP) images of the chest, upper abdomen, and lower abdomen, respectively (left to the right); **(B)** PET images; **(C)** Fused PET/CT images; **(D)** CT images.

**Figure 3:**
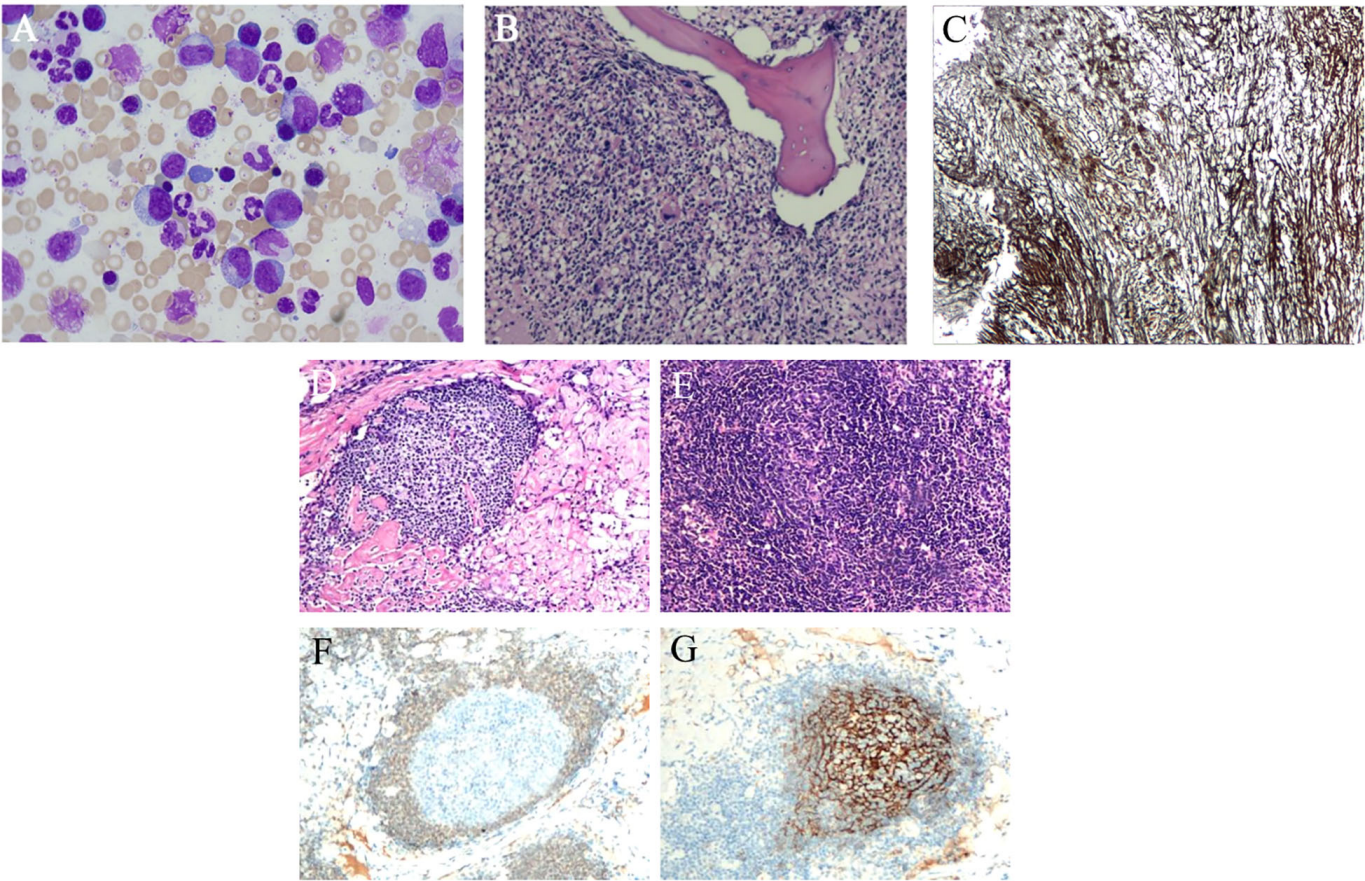
Bone marrow and lymph node pathology **(A)** Bone marrow image shows megakaryocyte dysplatelet production (HE staining). **(B)** Bone marrow pathology revealing marked fibrous tissue hyperplasia, a normal granulocyte-to-nucleated red blood cell ratio, and an increase in naïve cells. Numerous megakaryocytes with lobulated nuclei are present, along with scattered mature plasma cells and lymphocytes (HE staining). **(C)** Reticular fibers (MF-2) are highlighted with special staining. **(D)** Axillary and cervical lymph node pathology reveals increased interfollicular blood vessels that penetrate the germinal center, creating a “lollipop”-like appearance (H&E staining). **(E)** The mantle zone expands into a centripetal ring structure resembling “onion skin” (H&E staining). **(F)** The germinal center is negative for Bcl-2 staining. **(G)** The FDC meshwork forms a centripetally expanding spherical net (CD21 staining).

The patient had chronic multi-organ injury of unknown cause, then the ^18^F-FDG PET/CT scan were finally conducted and revealed findings suggestive of a proliferative disease of the lymphatic system, and hypermetabolic lymph nodes were detected in the neck, axillae, mediastinum, and hilar regions. Additionally, degenerative changes were found in several vertebral bodies (**Figure 2**). A lymph node biopsy should be attempted in order to exclude the cause of lymphoproliferative disorders, and finally the lymph node biopsy demonstrated characteristic features of hyaline vascular Castleman disease. Due to the changes of vertebral in ^18^F-FDG PET/CT, the bone marrow examination were conducted to differentiate various hematopoietic diseases and resulted in a dry tap which indicated myelofibrosis and megakaryocytic hyperplasia with atypia (**Figure 3**). Autoimmune, lymphatic and chronic infectious diseases could not be excluded. Then further investigations, including a serum free light chain assay, serum immunofixation electrophoresis, anti-ENA-like antibody spectrum, human herpesvirus 8 (HHV-8) DNA, cytomegalovirus DNA, Epstein-Barr virus (EBV) DNA, and metagenomic next-generation sequencing of ascitic fluid, revealed no significant abnormalities.

**Figure 4:**
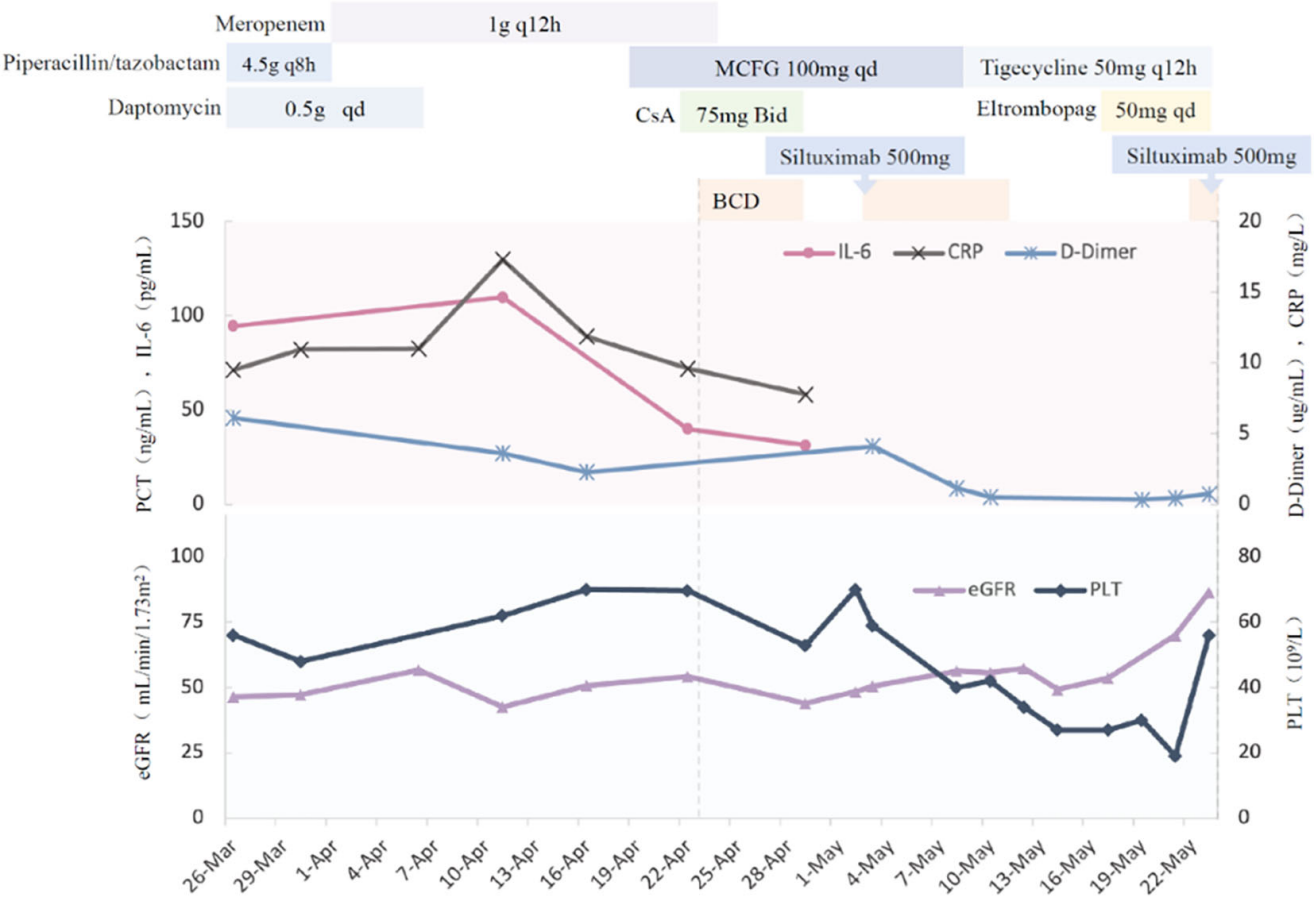
The detailed diagnosis and treatment timeline. BCD, Bortezomib, Cyclophosphamide, Dexamethasone.

According to “The Consensus on the Diagnosis and Treatment of Castleman Disease in China (2021)” the patient was diagnosed with TAFRO syndrome (iMCD-TAFRO subtype). A combination therapy regimen consisting of bortezomib, cyclophosphamide, dexamethasone, and siltuximab were conducted. Then we found that IL-6 levels significantly decreased, indicating treatment efficacy. However, chemotherapy was terminated due to myelosuppression, necessitating aggressive corrective measures. After hematologic recovery, the fourth dose of bortezomib and the second dose of siltuximab were administered. Following completion of the initial chemotherapy phase, the patient was discharged with the prescription of long-term oral thalidomide. The detailed diagnosis and treatment timeline is illustrated in **Figure 4**. Notably, five days post-discharge, the patient developed renal, respiratory, and circulatory failure secondary to severe abdominal infection. Despite intensive treatment, the patient ultimately passed away.

## Discussion

This study highlights a rare case of a patient with long-term abdominal distension, who was initially misdiagnosed as liver cirrhosis for over a year, while he was ultimately diagnosed as serious iMCD-TAFRO (2). TAFRO syndrome is identified as a systemic inflammatory disease, which mainly leads to a lack of recognition among non-hematologists. The diverse symptoms contribute to the patient being treated by multiple departments, elevating the risk of both misdiagnosis and missed diagnosis. The annual incidence of TAFRO syndrome in Japan was estimated to range from 0.9 to 4.9 cases per million, with fewer documented domestically and one-third of patients dead during first 24 months following diagnosis (3, 4). The present case was diagnosed with severe iMCD-TAFRO and exhibited poor outcomes despite aggressive treatment. This highlights the critical need for timely diagnosis and effective treatment. At present, there is no clear evidence on how to effectively recognize multiple symptoms of iMCD-TAFRO. Early detection, accurate diagnosis, and timely, severity-based treatment are crucial for extending patient survival. Further research is necessary to understand the underlying mechanisms and develop standardized treatment protocols for these patients.

TAFRO syndrome is classified as a subtype of idiopathic multicentric Castleman disease (iMCD) (1). The iMCD is often sub-classified into iMCD-TAFRO and iMCD-NOS, which is typically associated with chronic clinical course. The iMCD-TAFRO patients tended to be younger than iMCD-NOS who has an acute or subacute onset, and the general condition usually deteriorates quickly. Thus, life-threatening multi-organ and hospitalization burdens are more common in TAFRO syndrome, the same as this patient. Another conditions is that polyclonal thrombocytosis and hypergammaglobulinemia as definitively seen in iMCD without TAFRO, that is rarely observed in TAFRO syndrome (5). The megakaryocytic hyperplasia may represent a response to severe thrombocytopenia, which is likely due to peripheral platelet destruction. Alternatively, it is possible that the thrombocytopenia could be secondary to dysfunctional megakaryocytes, as megakaryocytic atypia was noted in most cases. In bone marrow pathologically, some TAFRO syndrome cases reticulin fibrosis was not noticeable in the bone marrow (6). Therefore, we should consider not only the presence of reticular fibrosis but also increases in the numbers of megakaryocytes and nuclear atypia as the features of TAFRO syndrome. This patient additionally exhibited an increased number of megakaryocytes, nuclear atypia, and reticular fibrosis, all of which possessed distinctive characteristics.

Lymph nodes histopathologically in TAFRO syndrome share features with iMCD, making differentiation challenging. There are three types of pathological: hypervascular, plasma cell and “mixed”, and TAFRO syndrome have hypervascular lymph node histopathologic features more frequently than iMCD-NOS. Iwaki’s criteria for TAFRO syndrome emphasize specific lymph node characteristics, including germinal center atrophy, enlarged endothelial nuclei, interfollicular endothelial vein hyperplasia, and a paucity of mature plasma cells. In contrast, Masaki’s criteria consider lymph node pathology as one of four secondary diagnostic criteria, making histologic confirmation less essential (7). In Japan, diagnosis of TAFRO syndrome can be made even in the absence of definitive histologic findings, particularly in cases marked by generalized edema, thrombocytopenia-induced bleeding, and minimal lymph node enlargement. Although lymph nodes in TAFRO syndrome tend to be small, PET/CT can detect abnormalities, which can then be confirmed via biopsy. Diagnosing iMCD-TAFRO remains challenging due to the lack of specific biomarkers, limited understanding of its pathophysiology, rapid disease progression, severe thrombocytopenia, and subtle lymphadenopathy. Early recognition of clinical features, identification of atypical symptoms, and preservation of pathological evidence are essential for accurate diagnosis.

The etiology and pathogenesis of TAFRO syndrome remain poorly understood. Recent research demonstrated that the condition involves cytokine dysregulation and autoimmune dysfunction triggered by multiple factors (8). The liver, being the largest immune organ, is often affected by TAFRO. A patient developed cirrhosis secondary to persistent fever and hepatosplenomegaly, accompanying by definitive diagnosis of TAFRO syndrome confirmed through histopathological analysis of liver lymph nodes. The condition was successfully managed with prednisone and tocilizumab (9). Additionally, a recent case report indicated the development of TAFRO syndrome following liver transplantation in a patient initially diagnosed with decompensated cirrhosis (10). This implies that inflammatory and lymphoproliferative diseases should be considered in cases of unexplained liver conditions like cirrhosis. Renal involvement is frequently found in iMCD-TAFRO, with most renal biopsy cases showing thrombotic microangiopathy or membranoproliferative glomerulonephritis -like lesions. In some cases, membranous nephropathy is also noteworthy, likely resulting from reduced glomerular perfusion and acute tubular injury due to microcirculatory disturbances (11, 12). These findings highlight renal hypoperfusion and ischemic tubular damage as major contributors to renal pathology in this condition (13, 14). In TAFRO syndrome, overactivation of B-cell promotes plasma cell expansion and polyclonal hypergammaglobulinemia. Autoantibodies and cytokines produced by specific B-cell clones suggest that iMCD, including TAFRO syndrome, exhibits autoimmune characteristics (15). TAFRO syndrome may be also associated with bacterial translocation that initiates a cytokine storm and drive systemic inflammatory responses (16). According to this patient’s clinical presentation, the pathogenesis of TAFRO syndrome involves: autoimmune mechanisms, systemic inflammation, and occult infections lead to increased vascular permeability, perfusion abnormalities, and megakaryocyte dysfunction, which in turn result in liver and kidney injury, thrombocytopenia, and associated clinical symptoms.

While the pathophysiology of iMCD and TAFRO syndrome remains not fully understood, the proinflammatory cytokine interleukin-6 (IL-6) is both a central driver of disease pathogenesis and a key therapeutic target (17, 18). Specifically, IL-6 exhibit a positive correlation with iMCD symptomology and progression, and inhibition of IL-6 serves as the recommended treatment for it (18). In this case, the treatment regimen included siltuximab, an IL-6 receptor antagonist, which significantly reduced cytokine levels post-treatment. Also, three classical signaling pathways: JAK/STAT3, PI3K/AKT/mTOR, and NF-κB is identified of great importance. Strategies that inhibit IL-6 and other pathways, have been noted for their effectiveness in iMCD-TAFRO syndrome. Furthermore, T-cell activation and regulation play a pivotal role in the pathogenesis of TAFRO syndrome. The PD-1 pathway, which induces T-cell exhaustion, has emerged as a potential therapeutic target (19). Cyclosporine suppresses helper T cells and CD8^+^ lymphocytes, thereby influencing T-cell proliferation and differentiation, that demonstrated efficacy in TAFRO syndrome. Additionally, glycogen synthase kinase 3β (GSK3β) and CC chemokine receptor 6 (CCR6) have been identified as upstream positive regulators of TAFRO syndrome, which could serve as both diagnostic markers and therapeutic targets for TAFRO syndrome (20). TAFRO syndrome frequently coexists with infections, malignancies, or autoimmune diseases. Some researchers demonstrated that TAFRO represents a severe variant of Sjögren’s syndrome, given shared immunological features, cytokine signaling abnormalities, and iMCD-like lymph node histopathology. This potential pathophysiological link may help refine clinical treatment strategies (21) Consequently, corticosteroids, rituximab, cytotoxic agents, immunomodulators, and anti-IL-6 therapies remain viable treatment options for iMCD-TAFRO. Early detection and timely intervention are critical for improving patient outcomes. Future research should prioritize identifying and managing the hyperinflammatory state associated with TAFRO syndrome to optimize therapeutic strategies. Given TAFRO syndrome characterized by numerous complications, ineffective treatments and poor prognosis, individualized treatment plans remain the key concentration for future exploration.

## Conclusion

TAFRO syndrome presents with a diverse range of symptoms, its underlying causes and mechanisms remain unclear, and treatment strategies are still being refined. There is a notable absence of effective treatment modalities. In the case presented, the patient exhibits involvement of the liver, kidneys, bone marrow, lymph nodes, and intestines, which are non-specific manifestations that can easily lead to misdiagnosis. Further clinical and pathophysiological research is essential to deepen the understanding of disease progression, improve diagnostic accuracy, optimize treatment approaches and enhance patient outcomes through early and timely intervention.

## Data Availability

The raw data supporting the conclusions of this article will be made available by the authors, without undue reservation.
